# Repetition of suicide attempts across episodes of severe depression Behavioural sensitisation found in suicide group but not in controls

**DOI:** 10.1186/1471-244X-11-5

**Published:** 2011-01-07

**Authors:** Louise Brådvik, Mats Berglund

**Affiliations:** 1 Department of Clinical Sciences Lund, Division of Psychiatry, Lund University Hospital, Lund, Sweden; 2 Department of Clinical Alcohol Research, University Hospital MAS, Malmö, Lund University, Sweden

## Abstract

**Background:**

Those who die by suicide and suffer from depression are known to have made more suicide attempts during their life-span as compared to other people with depression. A behavioural sensitisation or kindling model has been proposed for suicidal behaviour, in accordance with a sensitisation model of depressive episodes. The aim of the present study was to test such a model by investigating the distribution of initial and repeated suicide attempts across the depressive episodes in suicides and controls with a unipolar severe depression.

**Method:**

A blind record evaluation was performed of 80 suicide victims and controls admitted to the Department of Psychiatry between 1956 and 1969 and monitored to 2010. The occurrence of initial and repeated suicide attempts by order of the depressive episodes was compared for suicides and controls.

**Results:**

The risk of a first suicide attempt decreased throughout the later episodes of depression in both the suicide group (p < .000) and control group (p < .000). The frequencies of repetition early in the course were actually *higher *in the control group (p < .007). After that, the risk decreased in the control group, while the frequencies remained proportional in the suicide group. At the same time, there was a significantly greater decreased risk of repeated attempts during later episodes in the control group as compared to the suicide group (p < .000). The differences were found despite a similar number of episodes in suicides and controls.

**Conclusion:**

Repeated suicide attempts in the later episodes of depression appear to be a risk factor for suicide in severe depression. This finding is compatible with a behavioural sensitisation of attempts across the depressive episodes, which seemed to be independent of a corresponding kindling of depression.

## Background

Mood disorder is the single diagnosis with the greatest impact on suicide. In reviews of psychological autopsies it was concluded that an average of around 50%, 43% or 44% of all suicide victims had previously suffered from a depressive disorder [1-3].

Among depressed patients, suicide attempt is known to be a strong predictor for suicide [4-8]. Attempted suicide has been shown to be more likely when there are a higher number of depressive episodes [9] or more time spent in depression [10]. Furthermore, it has been concluded that once a suicide attempt has occurred, the patient is at high risk of more suicide attempts if future depressions occur [11].

Over the long-term course of depression, the onset of depressive episodes may become increasingly autonomous and less related to life-stressors [12,13]. This pattern has been hypothesised to result from a sensitisation process analogous to an animal electrophysiological model called "the kindling hypothesis" [14-16], or a behavioural sensitisation where every new episode gives rise to negative thinking patterns [17,18].

Those models may be applicable to suicidal behaviour as well as depression, and a cognitive processing for suicidal behaviour has been proposed [19]. To some extent, this proposal was indirectly supported by a cross-sectional study, which showed that patients with only one previous suicide attempt showed a significant correlation between intensity of suicidal ideation and life stress within 12 months, while patients with multiple self-harm showed no such relationship [20]. In other words, suicidal ideation appeared to be independent of life-stressors in the case of multiple self-harm. Furthermore, apart from death wish, an acquired ability to enact lethal self-injury has been proposed as a precursor of serious suicidality [21]. Number of past suicide attempts have been shown to predict acquired capability of lethal self-injury [22] in agreement with this proposal. Other investigators have found number of suicide attempts associated with a greater severity of suicidal symptoms [23]. Also, one has proposed that the painful and fear-inducing qualities of suicidality may diminish with repetition, whereas opponent processes (e.g., calming and pain-relieving effects) may intensify [24], and people may engage in more and more extreme behaviour [25]. Other authors have found that those who had both planned and attempted suicide were more impulsive than those who made suicide attempts without prior planning [26]. This indicates that impulsivity may be a mediator of suicide attempt by increasing the capability of making suicide attempt. In contrast however, greater lethality of current suicide attempt was not significantly associated with number of attempts in one study [23]. In addition according to that study, there was no reduction of pre-attempt stress, as has been suggested in the kindling theory of suicidal behaviour. However, none of these studies was a longitudinal investigation into suicidal behaviour across the depressive episodes, and so there was no direct evidence of a behavioural sensitisation. Furthermore, to our knowledge, no previous study has examined a possible sensitisation of suicide attempts in relation to fatal suicidal behaviour.

We have previously shown that suicide attempt predicts suicide in severe depression independent of severity, violence or repetition of the attempt [7]. This difference was found despite the finding that there were high and similar rates of adequate antidepressant treatment and also improvement across the episodes in those who died by suicide and controls [27]. People who died by suicide and suffered from a unipolar depression appeared to make suicide attempts across the later episodes more often than controls, while those with a bipolar disorder showed no significant difference in rates of suicide attempts across the episodes between those who died by suicide and controls [28].

The aim of the present study was to investigate the occurrence of initial and repeated suicide attempts during different depressive episodes in those who died by suicide and controls with a unipolar severe depression. A behavioural sensitisation model would imply that suicide attempts would be repeated throughout the episodes. This was hypothesized to occur in the suicide group, but not in the control group.

## Materials and methods

### The sample

**Figure 1:**
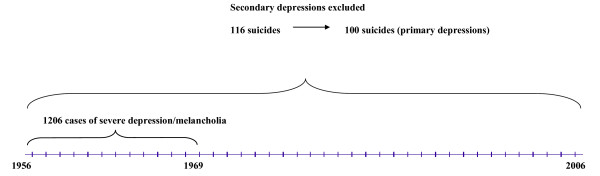
**Flow diagram for the sample of patients with severe depression admitted to the Department of Psychiatry, Lund University Hospital**.

In the 1950 s and 1960 s, all in-patients at the Department of Psychiatry, University Hospital, Lund were rated on a multiaxial diagnostic schedule at discharge [29]. This database enabled patients to be selected with a prospectively rated severe depression/melancholia for an investigation into suicide. The design of the sampling procedure is presented in a flow diagram (Figure 1). The very long-term follow up (to 2010) enabled a fairly high number of deaths by suicide to be investigated.

A total of 1,206 patients received the diagnosis severe depression/melancholia (506 men and 700 women). Their mortality was followed-up in three sessions: to January 1, 1984 to January 1, 1998, and to February 15, 2010. There were 116 suicide victims up to 2010. Out of these 103 had taken their lives up to 1984, another 11 up to 1998, and 2 more up to 2010.

The case records of those who died by suicide and matched controls from the total sample [30] were evaluated in detail. The researcher was unaware of the suicidal outcome and a similar procedure was carried out at second and third follow-up. A blind procedure allowed us to avoid the usual bias inherent in the retrospective evaluation. Secondary depressions were excluded according to Research Diagnostic Criteria [31], mainly alcoholism. Though alcohol dependence is related to a high risk of suicide [32,33], and is a major contributor to the suicide population [34,35], we excluded patients with primary alcohol dependence in order to study the contribution of depression alone on the suicidal outcome.

We obtained 100 deaths by suicide, 44 men and 56 women, with a primary severe depression. Matched controls, one for each suicide, were selected (from the total sample of 1,206 former in-patients of the Department of Psychiatry) using the criteria of diagnosis, gender, year of birth, and index admission year. The controls were chosen to be alive at the suicide death of the persons they matched and were monitored up to the time of death, so the length of follow-up was the same for both suicides and controls.

A retrospective diagnosis according to DSM-IV [36] was performed, based on the symptoms reported in the records. It turned out that 91% of the patients met the criteria for major depressive disorder with melancholic or psychotic features when in a depressive phase. Though the case-records were carefully written and very informative, individual symptoms might have been underreported, so the actual number was probably higher. Both the suicide group and the control group contained 20 patients who, at some time, had at least one episode of elevated mood, indicating bipolarity. There were 57 suicides and 57 controls that had an episode of psychotic depression at some time.

In the present study only the 80 suicide victims and 80 controls with a unipolar depression were investigated, as there had been no difference between suicides and controls in the decrease in suicide attempt rates in the bipolar group [28]. Though those with unipolar depression were not originally matched, they showed a similar age at index admission. There were 35 men in the suicide group and 36 in the control group and 45 and 44 women respectively in those groups.

### Suicide attempts

Suicide attempt was first scored by severity on the basis of the schedules introduced by Motto [36] and Weisman [37], as described in two previous papers [7,30].

We used a rather broad definition of self-harm, including what Motto [36] called suicidal gestures, cases where intent was difficult to determine on the basis of case records. The study started in 1984 and the same definitions were used in the two follow-ups in 1998 and 2010. Some more recent investigators also use a broad definition of self-harm without considering the degree of intent [39-41], which would include suicidal gestures and probably some aborted attempts (here ambivalent attempts). The latter have been described by Marzuk et al. [42] and have been associated with actual suicide attempts [43].

In the present sample, suicide attempt has, not unexpectedly, been found to be more common in the suicide group (46/80 versus 25/80), as reported before [28]. However, neither severity nor violence of method discriminated between those who died by suicide and controls [7]. (In the 2010 follow-up, 33% of the individuals in the suicide group sometimes made severe attempts as opposed to 28% in the control group; 43% and 52% respectively made violent.) Consequently, we chose to include all suicide attempts in the analysis regardless of severity and violence.

### Course of depression

The entire course of depression up to the deaths by suicide and a corresponding date for the matched control was evaluated. Those, who died by suicide and controls, both showed similar rates of episodes; an average of 3.88 (+/-3.44) episodes for those who died by suicide and 3.76 (+/-3.83) in the controls, and a median of 3 in both groups. It should be noted that the controls were not monitored after the suicide death they matched, and therefore the number of episodes in controls are compared for a certain time span and not for a life-time, so they may have more episodes later. (During follow-up of the total sample to 2010 none of the controls had died by suicide.) Treatment of depressive episodes was similar throughout the course of depression in those who died by suicide and controls, and so was improvement on treatment [27].

The study was approved by Lund University Medical Ethics Committee - 1985 and 2003.

### Statistics

Poisson regressions of the number of suicide attempts (per person) as a function of episode number and group (suicide deaths versus controls) was performed, where the decrease by higher episode number may be different for suicide deaths and controls. The differences of the initial level were also calculated [44]. Pearson's chi-square test was used for comparisons between groups [45].

## Results

### Repetition of suicide attempts

In the suicide group, as mentioned above, 46 patients had made suicide attempts (21 men and 25 women) compared with 25 patients in the control group (11 men, 14 women). Of these, 46% in the suicide group were repeaters compared with 40% in the control group. The average number of suicide attempts was 2.24 (SD +/- 2.77) in the suicide group and 2.32 (SD +/-3.61) in the control group

### Initial and repeated suicide attempt related to episode number

Suicide attempts were separated into initial and repeated attempts. There was no significant difference between suicide deaths and controls in rates of suicide attempt during the first episode.

**Figure 2:**
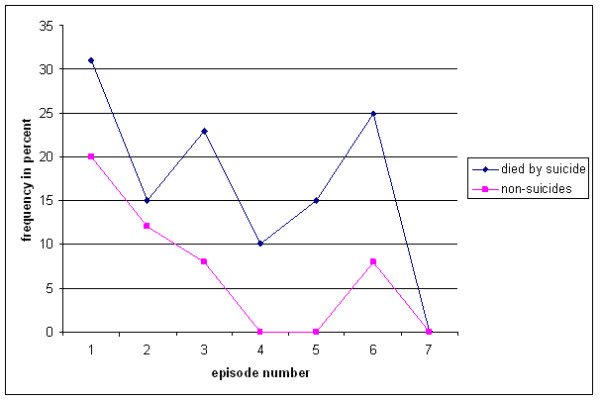
**Occurrence of initial suicide attempt by episode in suicides and controls**.

The risk of a first suicide attempt decreased throughout the later episodes of depression in both suicide deaths (p < .000) and controls (p < .000). No first suicide attempt occurred after the sixth episode in either group (Figure 2).

**Figure 3:**
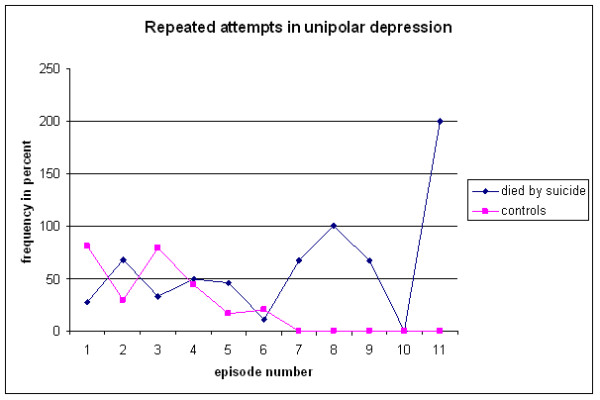
**Occurrence of repeated suicide attempts by episode in suicides and controls**.

The difference in suicide attempts during the course of depressive episodes was found among repeated attempts (Figure 3). The frequencies of repetition early in the course were actually *higher *in the control group (p < .007). After that there was a decreased risk in the control group, while the frequencies remained proportional in the suicide group. Consequently, there was a significantly lower risk of repeated attempts during later episodes in the control group as compared to the suicide group (p < .000).

## Discussion

### Main findings

Repetition of suicide attempts throughout the course of depressive episodes was more common among those who died by suicide as compared with those who did not. Two models for the development of a progressive behavioural dysfunction in the course of mood disorders have been proposed: behavioural sensitisation and kindling [14-19]. Such models might explain the fact that those who later die by suicide appear to continue to make suicide attempts after their first attempt throughout the course of depressive episodes. To the best of our knowledge, the present study is the first to give clinical evidence of the hypothesised behavioural sensitisation of suicide attempts [19]. Furthermore, the difference between suicide deaths and controls indicates that the behavioural sensitisation or kindling of suicide attempts is related to a suicidal outcome.

However, early in the course, controls had shown higher rates of repetition. In a previous paper we have shown that repeated suicide attempts in the controls were related to external stressors [7]. This may explain the finding that repetition was more frequent early in the course in controls, as repeated attempts may occur as a reaction to life-stressors and cease for some people when the crisis is resolved. On the other hand, the continuation of repeated suicide attempts in the suicide group could perhaps be described as a behavioural sensitisation or kindling phenomenon.

Previous studies have shown a positive correlation between number of episodes of depression and occurrence of suicide attempt [9,10,46]. Those findings may indicate that suicide attempts are likely to occur throughout the course of depressive episodes. In a previous study we found more episodes to be a risk factor for suicide only if these were associated with suicide attempts [28], and that the difference was found in the *unipolar *group only in contrast to the bipolar group. In the present study, however, we found that only *repeated *attempts occurring throughout the depressive episodes in the unipolar group discriminated between suicide deaths and controls. On the other hand, no first suicide attempt occurred after the sixth depressive episodes, a fact that does not support the view that spending more time depressed increases the risk for a suicide attempt.

As mentioned above, this development of suicidal behaviour was found despite the fact that suicide deaths and controls showed a similar number of episodes. In other words there was no corresponding increase in number of episodes in the suicide group as compared to the control group. There were also similar rates of adequately treated episodes, as well as improvement, in both groups. Consequently, the difference does *not *appear to be secondary to a more severe course of depression with more frequent episodes in the suicide group, *or *secondary to less adequate treatment.

To sum up, we have found clinical evidence for a behavioural sensitisation of suicidal behaviour. This is similar to the long-postulated kindling of depressive episodes [14]. However, the behavioural sensitisation appeared to be independent of the course and treatment of depression and may be a phenomenon for suicidal behaviour on its own.

### Clinical implications

Repeated suicide attempts in the later episodes of depression appear to be a risk factor for suicide in severe depression. Those who repeat in the later course should be treated with extra care.

### Strengths and limitations

The present study was based on a fairly large sample of patients with a severe depression/melancholia, who had been rated on a multiaxial schedule at their first admission with this diagnosis and monitored for 37-50 years. The number of deaths by suicide was fairly high, 80 with a unipolar depression. The agreement of diagnostics with DSM-IV appeared to be high, with at least 91% fulfilling the diagnostic criteria for major depressive disorder with melancholic or psychotic features. Only primary depressions were included, while depressions secondary to other disorders (mainly alcoholism) were excluded. As no depression was secondary to alcohol abuse, the impact of alcohol abuse was diminished.

The fact that the sample constitutes patients with a severe depression makes it less representative of a general sample of depressed patients. However, these patients seem to be at a particularly high risk of suicide [47] and also appear to predominate among suicide deaths [34], and therefore they are worth studying.

The definition of suicide attempt was based on two old papers [37,38], as the study started in 1984. This would correspond to suicidal acts with intent to die with and without injuries according to more modern definitions [40,41,48]. However, suicidal gestures according to Motto were also included. Such were for instance ingestion of a smaller amount of pills, where intent to die was not clearly stated (but would account as self-injury, as defined by O'Carroll - 48) or fetching a rope threatening to put around one's neck. Severity of attempt showed no correlation with fatal outcome, and therefore we included suicidal gestures in our analysis.

There were no personal interviews but only reports based on the case records. On the other hand, the suicide attempts have been continuously registered, thus minimising the recall bias inherent in interviews later in life. However, there is always a risk that some suicidal behaviour is never reported if there is no need for medical intervention. The crucial point is whether reports of repetition and severity are equally reliable for suicide deaths and controls. This could be assumed but not proven. The evaluation of the number of episodes was based on a blind evaluation of case records. The data about remission, recovery, relapse, and recurrence was based on reports of clinical evaluations. Once more, the reports were made at the time, thereby limiting the risk of recall bias. Furthermore, though there may be some uncertainty of the exact start of a new depressive episode, we do know the time sequence, i.e. we do know which suicide attempts occurred later in the course independent of the onset of a certain episode.

## Conclusion

Repeated suicide attempts in the later episodes of depression appear to be a risk factor for suicide in severe depression. In contrast, controls made repeated attempts during the early course of depression.

The difference could *not *be considered to be secondary to a more severe course of depression, or due to a lack of treatment in the suicide group, but to a difference in suicidal behaviour itself.

The present study gives clinical evidence of a behavioural sensitisation or a kindling model of suicide attempt across the depressive episodes, independent of a corresponding kindling of depression. Furthermore, this sensitisation appears to be related to a suicidal outcome as it was found in the suicide group only.

## Competing interests

The authors declare that they have no competing interests.

## Authors' contributions

LB initiated the study, contributed to the design and drafted the manuscript. MB contributed to the design. Both authors read the manuscript.

## Pre-publication history

The pre-publication history for this paper can be accessed here:

http://www.biomedcentral.com/1471-244X/11/5/prepub
